# RSV Monitoring in Germany: A Critical Overview of Available Surveillance Systems

**DOI:** 10.3390/jcm14217487

**Published:** 2025-10-22

**Authors:** Lea J. Bayer, Christian Brösamle, Gordon Brestrich, Bahar Najafi, Christof von Eiff, Cornelia Hösemann, Holger Stepan, Gunther Gosch, Michael Wojcinski, Michael Abou-Dakn, Egbert Herting, Markus A. Rose, Martina Prelog, Rolf Kaiser

**Affiliations:** 1 Pfizer Pharma GmbH, 10117 Berlin, Germany; 2 AMS Advanced Medical Services GmbH, 80639 Munich, Germany; christian.broesamle@ams-europe.com; 3 ZEG Berlin GmbH, 10115 Berlin, Germany; 4 Berufsverband der Frauenärzte e.V., 80335 Munich, Germany; 5 Department of Obstetrics, University Hospital Leipzig, 04103 Leipzig, Germany; 6 Medical Faculty, Otto von Guericke University Magdeburg, 39120 Magdeburg, Germany; 7 Department of Gynecology and Obstetrics, St. Joseph Hospital Berlin-Tempelhof, 12101 Berlin, Germany; 8 Department of Pediatrics, University Children’s Hospital of Lübeck, 23538 Lübeck, Germany; 9 Center for Congenital Lung Diseases, Department of Pediatric Pulmonology & Allergies, Olga Hospital, 70174 Stuttgart, Germany; 10 Pediatric Rheumatology/Special Immunology, Department of Pediatrics, University Hospital Würzburg, 97080 Würzburg, Germany; 11 Institute of Virology, University Hospital of Cologne, 50935 Cologne, Germany

**Keywords:** RSV, surveillance, epidemiology, respiratory infections

## Abstract

Respiratory syncytial virus (RSV) is a leading cause of respiratory infections in young children, elderly people, and patients with underlying diseases. Solid data on its epidemiology and burden of disease are essential for the implementation of preventive strategies. This review provides for the first time a comprehensive overview on publicly available RSV surveillance resources in Germany. **Methods:** Public RSV surveillance systems in Germany were identified and, where possible, exemplary data was extracted to provide an overview of the scope of available data, their strengths and limitations. **Results:** German RSV surveillance systems provide data on both outpatient and inpatient incidence rates, age distribution, and seasonality. Germany’s public health institution, the Robert Koch Institute (RKI), documents RSV cases nationwide based on mandatory reporting. Further, sentinel surveillance by RKI captures outpatient RSV infections as well as severe hospitalized cases. Nationwide, data on inpatients is collected and reported by hospital discharge diagnostic codes. Additional surveillance systems (e.g., clinical-virology.net) provide data on RSV positivity rates stratified by age and gender. Regional surveillance efforts by ten German states provide data on the infection dynamics. Pediatric documentation of age distribution and severity of respiratory diseases via surveillance was initiated by the German Society for Pediatric Infectious Diseases. Reviewing all available sources and data underlines the high clinical burden, especially in infants and older adults during the winter season. **Conclusions:** Germany’s RSV surveillance systems on the national and regional level support the tracking of incidence rates and seasonal patterns. Notably, pediatric data collection is more thorough, yielding a more comprehensive dataset than that available for adults. Contextualizing reported incidence rates in light of prospective or modeling studies suggests that the official documentation of RSV cases—particularly among adults—is underestimated.

## 1. Introduction

Respiratory Syncytial Virus (RSV), a member of the Pneumoviridae family, is a negative-sense, single-stranded enveloped RNA virus that regularly causes seasonal epidemics [1]. It exists in two subtypes, RSV A and RSV B, with both subtypes typically cocirculating and one subtype—mostly RSV-A—dominating each year [2,3]. RSV replicates in the epithelial cells of the upper respiratory tract and can also infect the bronchioles and/or alveoli, leading to lower respiratory tract illness (LRTI) with conditions such as bronchitis, bronchiolitis, and pneumonia. Common clinical symptoms of RSV infection (RSVI) include, but are not limited to, fever, rhinitis, cough, dyspnea, apnea, and chest tightness [1]. Additionally, neurotopic infections may occur with seizures and other symptoms of the central nervous system [4,5]. As RSV is highly contagious and is transmitted at any age through contact with saliva, contaminated surfaces, or respiratory droplets, approximately 50–70% of all children contract it within their first year of life. Nearly all children have had at least one RSV infection by the end of their second year of life [6]. Even mild RSV infections during infancy are associated with an increased risk of childhood asthma [7]. While most RSVIs result in only mild, common cold-like symptoms, a significant proportion will cause acute and severe respiratory tract illnesses, especially in premature and young infants, chronically ill individuals, and elderly subjects. The latter face a particularly high risk of experiencing cardiovascular complications, such as myocardial infarction or stroke due to activation of the thrombotic system by systemic inflammation caused by RSV [8,9,10,11]. Known risk factors for severe RSVI include underlying medical conditions such as cardiopulmonary diseases, a compromised immune response, prematurity, and very young or old age [9,12,13,14,15]. Current treatment options for RSVI are limited to the management of symptoms. After infection, individuals do not develop long-term protective immunity, making reinfections common. RSVI thus presents a significant health risk and has a substantial financial impact on the health care system [16,17,18]. In June 2024, Germany’s Standing Committee on Vaccination (STIKO) recommended RSV immunization for all infants in their first RSV season using the monoclonal antibody Nirsevimab [19]. This was followed by the STIKO recommendation of RSV immunization for all adults from 75 years of age as well as for adults aged 60–74 years with increased risk for severe RSV infections due to certain co-morbidities. The authors of STIKO’s justification of its recommendation underline the underestimation of RSV in adults, emphasizing the need for a deeper understanding of the virus’ epidemiology and its impact on patients’ lives [20].

In Germany, several distinct nationwide or state-level public surveillance systems track RSVI dynamics. On the national level, the Robert Koch Institute (RKI) compiles data on RSV epidemiology [21,22,23]. In addition, the Federal Health Monitoring Information System (Gesundheitsberichterstattung des Bundes, GBE-Bund) compiles routine administrative data on RSV-related hospitalizations from nearly all hospitals in Germany and also provides official German Cause of Death statistics [24]. Similar data are collected in InEK data, including detailed information on hospital cases and treatments [25]. The German Society for Pediatric Infectious Diseases (DGPI) started collecting data on hospitalized RSV cases among children from children’s hospitals and pediatrics departments in October 2021 [26,27,28,29]. The RespVir project, which is carried out by the Clinical Virology Network, collects and processes diagnostic data on a variety of respiratory infections from a set of laboratories in Germany, Austria, and Switzerland [30].

This review provides the first comprehensive synthesis of publicly available monitoring systems on respiratory syncytial virus (RSV) epidemiology in Germany and systematically evaluates them against a set of relevant criteria. We explore the range of existing databases and surveillance structures, examining their underlying methodologies, data collection strategies, and reporting mechanisms. Particular attention is given to the accuracy, representativeness, and timeliness of the reported data, as well as the extent to which these sources can be reliably used for different purposes, such as clinical decision-making, public health surveillance, and policy planning.

Beyond a technical assessment, we also discuss the strengths and limitations of each monitoring system, highlighting gaps in coverage, potential biases, and opportunities for improvement. In doing so, this review sheds light on how these data sources complement or overlap with one another, and how they may be integrated to form a more coherent and responsive epidemiological picture of RSV in Germany. To illustrate this, we extracted exemplary data to provide an overview of RSV seasonality, hospitalization incidence rates, and burden of disease.

Our overarching aim is to equip practicing clinicians and public-health decision-makers with a structured and transparent overview of the available resources, including direct links to databases to facilitate access. By mapping the current landscape of RSV monitoring systems, we provide a foundation for tracking infection dynamics in real time, analyzing the course of past epidemic seasons, and developing more accurate predictions of future trends. Ultimately, this knowledge will support the efficient allocation of healthcare resources and inform strategies for the treatment, prevention, and long-term management of RSV and other respiratory infections.

## 2. Methods

Databases and surveillance systems covering RSV in Germany were identified by searching for publications reporting on RSV in Germany. For the compilation of publicly accessible databases/surveillance systems, we included publicly searchable databases with access to raw data as well as annually published surveillance reports. Excluded were datasets based on results from nonrecurring studies and those published in a single report or publication. Past surveillance systems that are no longer active at the time of our search (June 2024 and updated in November 2024) were also excluded. Further surveillance systems were identified by expert consultation.

For the identification of regional RSV surveillance systems, we performed searches by search engine and AI (Google, Bing, Copilot) in March 2025 to check every federal state in Germany using the search terms (ARE = akute respiratorische Erkrankung; acute respiratory disease), “ARE Surveillance”, “ARE Erfassung” (capture), “Erfassung Atemwegserkrankungen” and “Surveillance Atemwegserkrankungen”. When regular surveillance efforts were identified, we checked for the inclusion of RSV in the reported data.

In order to evaluate the scope of each database/surveillance system, we analyzed the databases/surveillance system for the following criteria (see Table 1 and Table 2):

- Geographic reach
- Included age groups and age stratification
- Information on manifestation of disease (pneumonia, bronchiolitis, bronchitis, lower respiratory tract infection, upper respiratory tract infection)
- Type of sampling (source, technique)
- Type of RSV diagnostic tools (e.g., bedside rapid antigen test, professional antigen test, PCR, serum antibodies)
- Information on health care sector: inpatient/outpatient
- Laboratory analysis of RSV Subtype
- Information on seasonality
- Time lag until data availability
- Frequency of updates (weekly reports/season reports/time lag until update in database)
- Risk of bias/limitations of the database

To obtain an overview of the depth and level of detail with which the disease burden and epidemiology of RSV in Germany can be described based on publicly available databases, the relevant data were extracted in an exemplary manner.

Due to the large heterogeneity of the data from the different sources, a metanalytical approach was not feasible.

Incidence rates are reported as RSV cases per 100,000 persons per year, using the size of the respective population as the denominator.

### 2.1. Data Evaluation

#### 2.1.1. Robert Koch Institute (RKI)

To gather information on RSV incidence rates and patient age, the SurvStat database [31] and the annual reports published by the RKI were analyzed. SurvStat collects cases from mandatory reporting, which was implemented for RSV only in Saxony between 2020 and July 2023. For the calculation of outpatient incidence rates, weekly reports across all age groups were used during the 2018/2019 seasons.

In July of 2023, Germany extended mandatory reporting of RSV cases across all German states. Cases are recorded in four case definition categories: (i) clinical and epidemiological criteria met, (ii) clinical and laboratory criteria met, (iii) laboratory criteria met, clinical criteria not met, and (iv) laboratory criteria met, clinical criteria undetermined, and reported in SurvStat [31]. In addition, case numbers of a reference definition consisting of the sum of all four case definition categories is reported in SurvStat and also in the RKI’s annual reports. Data can be displayed by calendar year, season, calendar week, or season week and is available in close to real time after the reporting has reached the health authorities.

Data from the hospital-based surveillance program (ICOSARI) is available in the weekly reports (https://influenza.rki.de/Wochenberichte.aspx; accessed on 11 January 2025) and has been reported in detail by Cai et al. for patients between 2009 and 2018 [22].

#### 2.1.2. Landesuntersuchungsanstalt (LUA) Saxony (Saxonian Health Institute)

Reported case numbers were used to calculate a population-based RSV incidence. Data on RSV cases were extracted from the annual reports for 2016 to 2021, which are available on the LUA homepage [32]. The annual population of Saxony for the respective years was used to calculate a population-based RSV incidence rate as the number of reported RSV cases per 100,000 persons per year.

#### 2.1.3. German Society for Pediatric Infectious Diseases (DGPI)

Data on children hospitalized with RSVI were selected and downloaded for the 2021/22, 2022/23, 2023/24 and 2024/25 seasons from the DGPI website [26,27,28,29].

#### 2.1.4. GBE-Bund (National Health Reporting of Germany)

The annual incidence of RSV inpatient cases per 100,000 persons was calculated using the 2011 German standard population for all age categories, as well as for infants under one year of age. Both primary and secondary diagnoses for these groups in the respective years were recorded using the ICD10 codes J12.1 “Respiratory syncytial virus pneumonia”, J20.5 “Acute bronchitis due to respiratory syncytial virus”, J21.0 “Acute bronchiolitis due to RSV”, and B97.4 “Respiratory syncytial virus as the cause of diseases classified elsewhere”.

#### 2.1.5. The German Institute for the Hospital Remuneration System (InEK)

As the InEK data browser sources from the same hospital ICD-10 codes as GBE-Bund, InEK data was not analyzed separately. A recently published study analyzed data from InEK to report the inpatient burden of RSV in children ≤2 years of age in Germany for 2019–2022 [18].

#### 2.1.6. RespVir/Clinical Virology Network

Monthly data of submitted RSV-positive samples were extracted from the year 2015 through May 2022 from the RespVir dashboard [33].

## 3. Surveillance Systems and Databases on RSV in Germany

### 3.1. Robert Koch Institute (RKI)

(1) The RKI compiles and presents in its national surveillance database (SurvStat@RKI) data from mandatory reporting of RSVI, both from the outpatient and inpatient sector. Between 2002 and July 2023, Saxony was the only state with mandatory reporting of laboratory-confirmed RSV cases. In July 2023, mandatory reporting was extended across all German states. For the 2023/2024 season, a total of 57,541 cases and for the 2024/2025 season (as of 20 May 2025) 68,265 cases were reported based on the RKI’s official “Referenzdefinition” (reference case definition). In more than 60% of accumulated cases from both seasons, both clinical and laboratory criteria were met, meaning a clinical respiratory infection of a patient was confirmed as RSVI by a laboratory test. Another 34% of recorded cases arose from a positive laboratory test that could not be correlated to a specific sick patient (clinical criteria undetermined). Cases that relied only on clinical and epidemiological evidence in the absence of laboratory data or cases in which a positive lab test was not accompanied by clinical signs of RSVI accounted together for less than 6% of all reported cases.
  As a centralized reporting system for notifiable diseases, SurvStat provides near real-time monitoring and thus supports outbreak detection. However, the data lacks clinical detail. Additionally, data availability depends on reporting compliance, making it vulnerable to underreporting.
(2) Physicians across Germany report current diagnoses of respiratory diseases in the outpatient sector to the online database called ARE Sentinel Surveillance. This data source consists of approximately 700 primary care practices as a representative sample of the population. The data collected include information on the severity and frequency of current RSVIs, specifically acute respiratory diseases, based on clinical symptoms. Additionally, virological surveillance is conducted in approximately 100 sentinel practices that submit patient samples of symptomatic patients to the National Reference Center to identify currently circulating respiratory viruses. The collected data is evaluated on a weekly basis and presented in the form of weekly reports [21]. Data on RSV consultation rates can be downloaded from an online repository [34] with an additional extraction step.
  The ARE Sentinel Surveillance offers broad insights into respiratory illness trends across age groups and regions in the outpatient sector by capturing syndromic as well as virological data. Although representative sampling is aimed for, the system is limited by its sample size (>1% coverage of primary care physicians in Germany) and regional differences in coverage.
(3) Nationwide, “citizen scientists” can self-report cases of acute respiratory infections (ARIs) at the population level to the online database known as “GrippeWeb”. Individuals aged 16 years and above, residing primarily in Germany, can participate through the web portal in a population-based approach. They can self-report once a week whether they experienced a new respiratory illness in the preceding week. This process tracks the percentage of the entire population that has developed an acute respiratory infection on a weekly basis, including visits to their general practitioners (GPs) [35].
  A strength of GrippeWeb is the estimation of ARI incidence even independent of healthcare utilization by collecting self-reported data. However, the data may be biased or incomplete due to self-reporting, and it lacks clinical and virological specificity. The latter limitation is addressed by “GrippeWeb-Plus”.
(4) Since 2020, the RKI has been conducting an additional virological surveillance program as part of GrippeWeb (influenza web), called “GrippeWeb-Plus.” In this program, a randomly selected sample of regularly reporting GrippeWeb participants receive swab materials. In the event of an acute respiratory infection, they take a sample from their frontal nasal area and subsequently send it to the RKI for testing for 24 different respiratory pathogens, including influenza viruses, SARS-CoV-2, and RSV. Currently, around 800 children and adults from approximately 480 different households participate in GrippeWeb-Plus. Since multiple individuals from one household participate in GrippeWeb-Plus, a household-adjusted positive rate is calculated [36].
(5) The syndromic surveillance of severe acute respiratory infections (SARIs) in the inpatient environment involves the use of ICD-10 codes to monitor cases in sentinel hospitals (referred to as ICOSARI, [23]). This surveillance is conducted in approximately 70 selected hospitals, covering about 5–6% of all hospitalized patients in Germany, and is considered representative. As ICOSARI is dependent on ICD-10 codes, it may be affected by coding inaccuracies.
(6) As of week 7, 2025, data on RSV detection in wastewater has been reported [37]. For the first report in February 2025, data from 25 wastewater treatment plants was analyzed and reported as viral load, stratified by RSV subgroup A and B [38]. Data provided by further wastewater treatment plants will be included subsequently. A key challenge lies in establishing standardized sampling frequencies and laboratory methodologies to ensure data comparability and reliability across surveillance sites.

**Table 1:** Key descriptors of RKI ARE and RSV Monitoring systems.

	SurvStat	ARE Sentinel Surveillance		GrippeWeb	ICOSARI
Descriptor		Consultation	Virological Surveillance		
**Who reports**	Physicians, laboratories, public health authorities	Physicians	Participating laboratories	Citizens	Physicians via ICD-10 coding
**Geographic reach**	Germany; stratification by state, territorial unit, and district	Germany	Reported for each German state, as well as Germany-wide	Germany	Germany
**Age stratification**	Fine-grained age stratification	0–1 y, 2–4 y, 5–14 y, 15–34 y, 35–59 y, >59 y	0–1 y, 2–4 y, 5–14 y, 15–34 y, 35–59 y, >59 y	0–4 y, 5–14 y, 15–34 y, 35–59 y, >59 y	0–4 y, 5–14 y, 15–34 y, 35–59 y, 60–79 y, >79 y
**Information on manifestation of disease**	No	Yes	No	Influenza-like illness (ILI) reported	Includes only severe acute respiratory infections
**Type of sampling and test**	Antigen test, PCR, or epidemiological confirmation	N/A	Usually nasopharyngeal swab; usually tested by RT—PCR [39]	N/A	N/A [22,23]
**RS-virus subtyping**	No	No	No	No	No
**Information on seasonality**	Yes	Yes	Yes	Yes, for ARE, not for RSV specifically	Yes, for ARE, not for RSV specifically
**Frequency of updates**	weekly	weekly	weekly	weekly	weekly
**Limitations/Risk of bias**	Includes only confirmed RSV cases. Testing frequency and quality varies substantially by patient age.	Syndromic reporting of patients with acute respiratory diseases; does not require testing.	Quality of sample material influences virus detection.	Convenient sampling, depending on voluntary reporting by citizens. Subjective self-reporting of symptoms. No testing for pathogens.	ICD-10 based surveillance; bias due to diagnosis- and coding-practice. Varying testing reported [22,23]

Abbreviations: RSV = respiratory syncytial virus, RKI ARE = respiratory disease monitoring by Robert Koch Institute, y = years of age, N/A = not applicable, RT-PCR = reverse transcription polymerase chain reaction,.

**Table 2:** Key descriptors of further ARE and RSV Monitoring systems in Germany.

Descriptor	DGPI [40,41]	ClinicalVirology [33]	GBE-Bund/InEK [25]	ARE Surveillance by Federal States
**Who reports**	Participating hospitals	Participating laboratories	All hospitals billing according to the DRG reimbursement system	
**Geographic reach**	Germany	Germany (plus Austria and Switzerland)	Germany	10 German Federal States (see Table 3)
**Age stratification**	0–3 months, 4–11 months, 1–2 y, 3–4 y, 5–11 y, 12–18 y, ≥19 y	0 < 6 y, 6 < 13 y, 13 < 19 y, 19 < 46 y, 46 < 65 y, ≥65 y	**InEK**: <28 days, 28 days—<1 y, 1–2 y, 3–5 y, 6–9 y, 10–15 y, 16–17 y, 18–29 y, 30–39 y, 40–49 y, 50–54 y, 55–59 y, 60–64 y, 64–74 y, 75–59 y, ≥80 y **GBE-Bund**: <1 y, 5 y increments	Depending on the state. E.g. Saxony-Anhalt: <2 y, 2–6 y, 7–17 y, 18–59 y, ≥ 80 y Often age stratified data not provided for RSV.
**Information on manifestation of disease**	All hospitalized cases. For 2021/22, 2022/23 and 2023/24 data for regular ward and ICU cases available.	No	Yes, by ICD-10 code	No
**Type of sampling and test**	Antigen test and/or PCR	Singleplex and Multiplex PCR	Not documented	For laboratory-based surveillance, respiratory samples collected by participating sentinel practices are most likely analyzed using PCR testing.
**RS-virus subtyping**	No	No	No	Usually not reported (exception Baden-Württemberg)
**Information on seasonality**	Yes, near real-time updates in the current season.	Yes, near real-time updates in the current season.	Yes (diagnoses provided by date, but with delay of several months)	Yes, near real-time updates in the current season.
**Frequency of updates**	Current case numbers provided on weekly basis.	Current case numbers and positivity rates provided on weekly basis.	The billing data in the InEK data browser is provided three times a year, on June 15th, October 15th, and January 15th. These data include discharges from January 1st to May 31st, January 1st to September 30th, and January 1st to December 31st of the current calendar year.	Weekly reports on current season.
**Limitations/Risk of bias**	Voluntary participation of hospitals	Voluntary participation of laboratories. In most settings, only patients with respiratory symptoms are tested.	Only ICD-10 coded diagnoses documented.	Usually preschool children as sentinels for virus activity.

Abbreviations: ARE = acute respiratory disease, RSV = respiratory syncytial virus, DGPI = German Society for Pediatric Infectiology, ICU = intensive care unit, ICD-10 = international statistical classification of diseases V.10, PCR = polymerase chain reaction.

**Table 3:** German Federal States that conduct additional ARE surveillance (as of March 2025).

Federal State	Independent ARE and RSV Surveillance	Notes
Baden-Württemberg	Yes https://www.gesundheitsamt-bw.de/aktuelles-und-service/newsletter-und-infodienste/are-bericht/ (last accessed on 14 October 2025).	Reports case numbers for RSV-A and -B separately
Bavaria	Yes https://www.lgl.bayern.de/gesundheit/infektionsschutz/molekulare_surveillance/bis_c/bisc_ergebnisse.htm (last accessed on 14 October 2025).	ARE surveillance, incl. RSV, established in 2009 (“Bayern Influenza Sentinel” (BIS)); wastewater monitoring for RSV introduced in 2025
Berlin	Yes https://www.berlin.de/lageso/gesundheit/infektionskrankheiten/berichte-veroeffentlichungen/wochenberichte/ (last accessed on 14 October 2025).	RSV reported since season 2023/24
Brandenburg	No	-
Bremen	No	-
Hamburg	No	-
Hesse	Yeshttps://hlfgp.hessen.de/gesundheitsschutz-gesundheitsdaten/gesundheitsdaten (last accessed on 14 October 2025).	RSV reporting established in September 2023
Mecklenburg Western Pomerania	Yeshttps://www.lagus.mv-regierung.de/Gesundheit/InfektionsschutzPraevention/ARE/ (last accessed on 14 October 2025).	-
Lower Saxony	Yes https://www.nlga.niedersachsen.de/are/uebersicht-205132.html (last accessed on 14 October 2025).	ARE surveillance, incl. RSV, established in fall 2004
Northrhine-Westphalia	Yes https://www.lzg.nrw.de/inf_schutz/meldewesen/infektionsberichte/wochen-infektionsberichte/index.html (last accessed on 14 October 2025).	ARE surveillance since season 2014/15; inclusion of RSV in season 2022/23
Rhineland Palatinate	Yes, link https://lua.rlp.de/unsere-themen/humanmedizin/daten-zu-atemwegserkrankungen/wochenberichte#c79568 (last accessed on 14 October 2025).	ARE surveillance established in 2023
Saarland	No	-
Saxony	Yes https://www.gesunde.sachsen.de/epidemiologische-berichte-4057.html (last accessed on 14 October 2025).	Mandatory reporting of laboratory-confirmed RSV cases since 2002
Saxony-Anhalt	Yes https://verbraucherschutz.sachsen-anhalt.de/gesundheit/wasserhygiene/trinkwasser-1/surveillance-akuter-respiratorischer-erkrankungen-are (last accessed on 14 October 2025).	ARE surveillance established in 2007; Representative sample of children aged 3 to 6 years
Schleswig-Holstein	No	-
Thuringia	No	-

ARE = acute respiratory disease (akute respiratorische Erkrankung).

### 3.2. Surveillance of ARE (“Akute Respiratorische Erkrankungen”, Acute Respiratory Illnesses) Surveillance by Federal States in Germany

The State of Saxony Ministry of Social Affairs was the first state to establish an ordinance that mandates the regional reporting of laboratory-confirmed RSV cases (both hospitalized and non-hospitalized) under the State Infection Protection Act [42] in 2002. The reporting is carried out by the testing laboratories.

The reported data is transmitted to the RKI by the Landesuntersuchungsanstalt (LUA; Saxonian Health Institute). Based on the infection epidemiological data, weekly and monthly epidemiological reports are prepared. Additional analyses and trend assessments are included in an annual report.

Several additional states have implemented surveillance systems to monitor Acute Respiratory Diseases via voluntary reporting. Some states have recently established ARE surveillance systems during the COVID-19 pandemic; these systems are now also covering RSV (see Table 3).

In summary, most states activate their ARE surveillance during the typical cold season when the risk of contracting cold-related illnesses is highest; approx. from the 40th to the 15th calendar week of the following year. In most cases, the surveillance comprises two components: a reporting system for ARE incidence in preschool day-care facilities and participation of (pediatric) sentinel practices. Regional ARE reporting complements national systems and supports localized outbreak detection and preparedness. Notably, substantial regional disparities exist, as no data on ARE reporting could be identified for six of Germany’s sixteen federal states (see Table 3). Federal Health Monitoring (Gesundheitsberichterstattung des Bundes, GBE-Bund).

The federal health reporting online database (GBE-Bund) is a service run by the German Statistical Office (DESTATIS) and serves as a central hub for consolidating health-related data and information from a wide array of more than 100 sources [24]. All hospitals that bill according to the DRG reimbursement system and fall under the scope of § 1 of the Hospital Remuneration Act (KHEntgG) are required to submit data. Main and secondary diagnoses originating from all reporting hospitals in Germany are recorded in an annual survey in the GBE-Bund database and compiled with administrative data, which is predominantly derived from coded discharge diagnoses based on the 10th International Statistical Classification of Diseases and Related Health Problems (ICD-10). Apart from information on hospital primary diagnoses, the database provides data categorized by the place of treatment, the patient’s age, gender, residence, duration of stay, and data on cause of death [43]. Additionally, for all codes, secondary diagnoses can be obtained through the GBE-Bund database on request. Based on administrative data from German hospitals, the GBE-Bund database constitutes one of the most comprehensive sources for estimating the burden of RSV and associated healthcare utilization across large population segments. However, the availability of data is subject to a considerable time delay.

### 3.3. German Institute for the Hospital Remuneration System (InEK)

InEK is the organization responsible for developing and maintaining the Diagnosis-Related Groups (DRG) system in Germany. The DRG system is used for hospital reimbursement, where patients are grouped based on their diagnoses and treatments. As required by law, case-related data are to be reported to InEK by every hospital in Germany. Data can be extracted via an online data access tool using primary and secondary diagnoses (ICD-10-codes), age groups, diagnosis-related groups, discharge reason, length of stay, ICU admissions, ventilation hours, and dates of admission and discharge as search categories [25]. Similar to GBE-Bund, InEK provides a comprehensive dataset covering the majority of German hospitals. However, the InEK online system offers enhanced filtering capabilities and greater data granularity. As with GBE-Bund, a key limitation is the delay in data availability.

### 3.4. German Society for Pediatric Infectious Diseases (DGPI)

As Germany experienced a surge of RSV cases in the post COVID-19 pandemic season in 2021/22, the DGPI initiated an ad hoc nationwide surveillance program with 156 participating German pediatric hospitals that monitored hospitalizations due to RSV among children [26]. For the RSV seasons 2022/23, 2023/24, and 2024/25, a registration system for respiratory tract infections that allows pediatric hospitals across Germany to record clinically significant respiratory infections that resulted in hospital admissions was introduced. The number of participating children’s hospitals and pediatric departments increased from on average 37 in 2022/23 to 51 in 2023/24 and to 61 (18.3% of pediatric hospitals in Germany) in 2024/25. The survey tracks the percentage of admissions to the hospital and to intensive care units attributed to RSV, SARS-CoV2, and influenza, providing fine-grained age stratification. The data is visualized online on the DGPI homepage [27,28,29]. The DGPI’s ‘AWI-Erfassung’ system monitors hospitalized pediatric respiratory cases with high data quality and timely updates. The scope of data collection is determined annually based on current needs. Initially limited to RSV cases in 2021/22, it was later expanded to include all respiratory illnesses. While data on both new admissions and intensive care were available for 2022/23 and 2023/24, only admission data is currently provided for the 2024/25 season. Tracking for 2024/25 additionally includes the proportion of children aged 0–2 years with RSV immunization or maternal RSV vaccination during pregnancy.

### 3.5. RespVir, Clinical Virology Network

RespVir was established in 2007 as an initiative originating from a clinical virology working group (now the Clinical Virology Network) is part of “Deutsche Vereinigung zur Bekämpfung der Viruserkrankungen” (German Society for Infectious Disease Control) and affiliated with the German Society for Virology (GfV), and the “Paul-Ehrlich-Gesellschaft für Infektionstherapie” (Society for Infectious Diseases Therapy). Its primary objective is to maintain an online database for the comprehensive documentation of respiratory infections, offering healthcare professionals close to real-time information about currently circulating pathogens.

The Clinical Virology Network (RespVir, CVN) database contains diagnostic data sourced from around 55 laboratories, mainly including university hospitals and private laboratories in Germany, Austria, and Switzerland.

The database analyzes 26 different respiratory pathogens, comprising 18 viruses and eight bacteria. RespVir is designed to incorporate data from samples collected from all patients exhibiting respiratory symptoms and submitted by physicians for diagnostic purposes. Most samples are from hospitalized patients. Subjecting the samples to mono- and multiplex PCR testing methods reduces the bias that may result from testing only for selected pathogens [44]. A distinctive feature of the CVN is that it collects not only positive RSV test results but also negative ones. This allows for the calculation of positivity rates, which provide a more accurate reflection of epidemiological trends, as absolute case numbers are heavily influenced by overall testing volume. An increase in the positivity rate can indicate rising infection levels, even when the absolute number of cases declines.

Reports on test results in the form of a dashboard regarding respiratory infections can be accessed through an online database interface [33]. Data can be visualized stratified by age and gender. A key strength is the ability to show results either as absolute counts or as positivity rate, which enables comparisons between periods of low test numbers and periods of high test numbers. Limitations include the non-population-based design, absence of detailed clinical data, and variable site participation.

Table 4 provides hyperlinks to publicly accessible surveillance systems and databases.

## 4. RSV Burden of Disease in Germany

### 4.1. RSV Epidemiology in Germany

#### Seasonality of RSV

**Figure 1:**
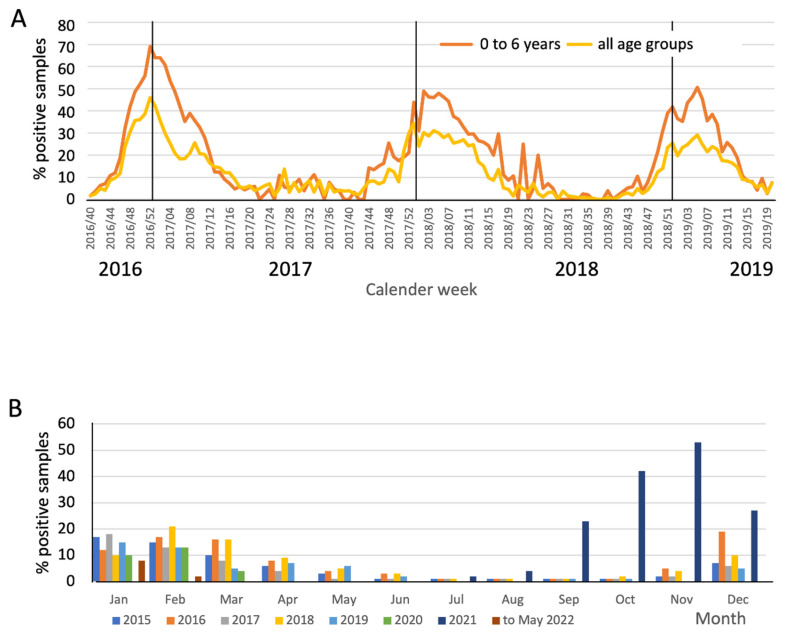
Seasonality of RSVI from 2016 to 2022: Percentage of positive samples. (**A**) RespVir program of Clinical Virology.Net with data of mostly hospitalized patients (**B**) RespVir data aggregated for the years 2015 to 2022. Note the atypical dynamics of the 2020/21 and 2021/22 seasons.

As they do in many countries in the Northern hemisphere, RSVIs display a typical seasonal pattern in Germany, with interruptions of the established seasonality observed thus far only during the COVID-19 pandemic (Figure 1). The RKI AG Influenza’s weekly reports for the years 2016–2019 reveal RSV seasons that span on average 14–18 weeks and typically peak during the winter months of January and February, usually starting between November and January [45]. This dynamic is very similar to the situation in hospitalized patients as reported through the RespVir program of the Clinical Virology Net (Figure 1A,B). During the RSV season, hospitalized children up to six years of age were more likely, compared to all age groups, to have a positive RSV test result. At the height of the 2018/19 season, 51% of all tests among children aged 0–6 years were positive for RSV, while the positivity rate for all age groups was substantially lower at 29%. Positivity rates of the tested samples aggregated across the months of the year show regular off-season/on-season patterns from 2015 to the beginning of 2020 and thus underscore the atypical course of the 2020/2021 and 2021/2022 seasons (Figure 1B). Most likely due to the increased non-pharmaceutical interventions (NPIs) during the SARS-CoV2 pandemic, RSVI (as well as respiratory infections due to other pathogens) was virtually absent from July 2020 to June 2021 [46]. In 2021, RSVI returned unexpectedly early with unusually high numbers of positives, which peaked in August [26,27].

### 4.2. RSV Incidence

**Figure 2:**
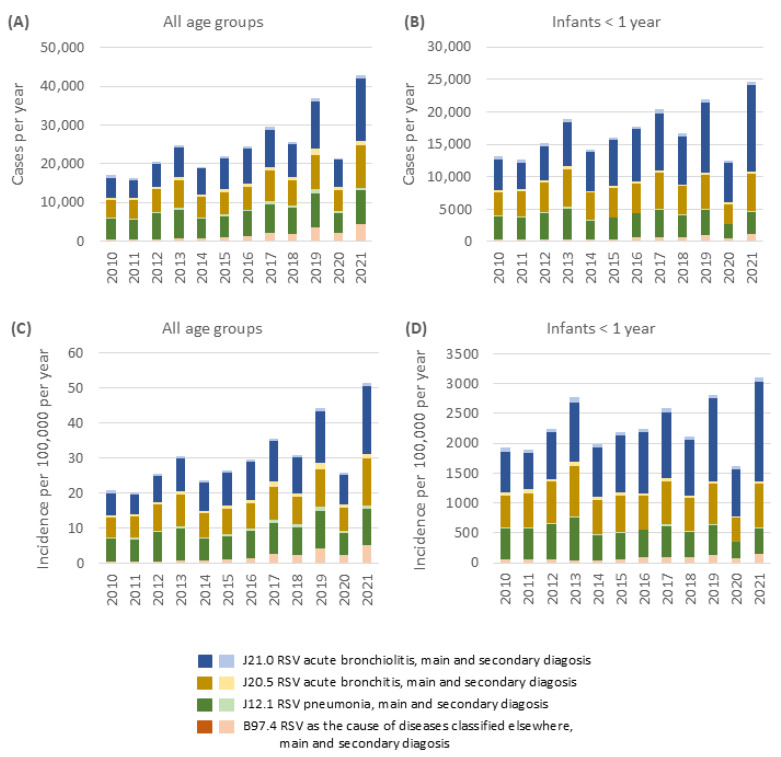
Hospitalization incidence for RSV infection in Germany 2010 to 2021: Administrative data by ICD10 codes. Case numbers across (**A**) all age groups and (**B**) in infants below 1 year of age. Population-based incidence per 100,000 for (**C**) all age groups and (**D**) for infants below 1 year of age.

To determine absolute numbers of clinically relevant RSVI cases and estimate their impact on the German health care system, data on the diagnoses of hospitalized patients of the German Federal Statistical Office for the years 2010 to 2021 were analyzed (Figure 2). Between 2010 and 2021, documented RSVI cases fluctuated between 16,169 (2011) and 42,857 (2021) per year, resulting in incidence rates from 20 to 51 cases per 100,000 per year (Figure 2C) across all age groups. Most of these cases occurred in children within the first year of life, with case numbers between 12,575 (2011) and 24,637 (2021), yielding incidence rates between 1898 and 3097 per 100,000 (Figure 2D). Bronchiolitis (ICD10-J21.0), bronchitis (ICD10-J20.5), and pneumonia (ICD10-J.12.1) had a significant share among RSVI. Most of the RSVIs in German hospitals were recorded as the primary diagnosis.

The incidence rate of outpatient visits to doctors’ offices for RSVI can be estimated from the sentinel program of the RKI. During the 2018/2019 season, between 800 and 2000 consultations per 100,000 were recorded in association with respiratory infections [47]. Differentiation by age showed that consultations for RSVI were strongly skewed toward young children: in infants up to 1 year of age, the average consultation incidence was 12,400/100,000 per year and thus about twice as high as the consultation incidence for influenza. In children aged 2 to 4 years, this value was still at 7700/100,000 per year. For children aged 5 to 14 years (1100/100,000), adolescents and young adults (15 to 34 years, 800/100,000), adults (35 to 59 years, 600/100,000), and the elderly (≥60 years, 700/100,000), yearly consultation incidences were considerably lower [47].

From 2016 to 2021, the incidence of RSVI notifications in Saxony among all age groups rose from 61/100,000 to ca. 144/100,000 (with a dip in 2020 due to non-pharmaceutical interventions during the COVID-19 pandemic) [32]. During the same period, the RSV hospitalization incidence among all age groups in Saxony, as recorded by the German Statistical Office, increased from 22/100,000 to 52/100,000 per year.

Nationwide, SurvStat recorded during the 2024/25 season (as of 20 May 2025) 68,265 cases, resulting in an incidence rate of approx. 78/100,000 across all age groups and an incidence of approx. 88/100,000 in >60-year-olds. Incidence rates were considerably higher in infants (1014/100,000 in <1-year-olds), small children (710/100,000 in <5-year-olds), and persons of old age (208/100,000 in >79-year-olds).

### 4.3. Severity and Hospitalization Burden of RSVI

**Figure 3:**
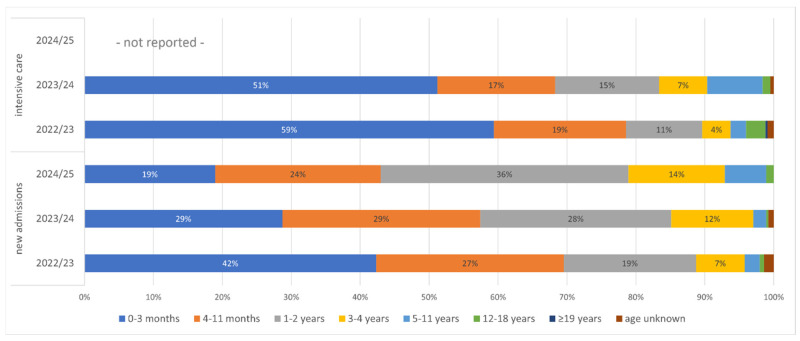
Ad hoc ARI surveillance by DGPI. Age distribution of patients hospitalized with RSV during the 2022/23 to 2024/25 seasons, stratified by intensive care vs. newly admitted patients.

The DGPI’s ad hoc RSVI surveillance for the 2021/22 season provides data on the average number of daily RSV cases reported from both regular hospital wards and intensive care units. Approximately 10% of hospitalized RSV cases required intensive care. The stratification by age provided for the seasons 2022/23, 2023/24 and 2024/25 again revealed a prominent overrepresentation of small children among patients hospitalized for RSVI, especially during the years following the end of the COVID-19 pandemic: between 58% and 69% of newly admitted patients and between 68% and 78% of patients in intensive care units were under the age of one year. In 2024/25, a similar number of infants in their first year of life and toddlers in their second year were admitted, accounting for 33% and 36% of all new pediatric admissions, respectively (Figure 3).

The ICD-10-code-based hospital surveillance study for severe acute respiratory infections (ICOSARI) of 8761 RSVI cases between 2009 and 2018 largely confirmed the high burden of severe RSVI among infants, with 57% of hospitalized RSVI patients < 1 year of age [22]. About 5.6% of admitted patients received intensive care and 38% of these required ventilator support. Twenty-five patients died, with almost half of them over the age of 65 years. The case fatality rate (CFR) of hospitalized RSVI cases varied significantly depending on the age of the patient. Of 122 severe RSV cases among the elderly aged 65 and older, 12 patients passed away, yielding a high CFR of 9.5%. Among the 7996 infants with severe RSV, there were 8 fatal cases, yielding a CFR of 0.1%. The examination of potential risk factors identified several underlying conditions that were more frequently found in severe cases. Notably, respiratory and cardiovascular disorders specific to the perinatal period as well as cardiovascular diseases were highly prevalent in patients receiving intensive care (13% and 14%) and even more so in patients that needed ventilator support (19% and 25%) [22].

## 5. Discussion

**Figure 4:**
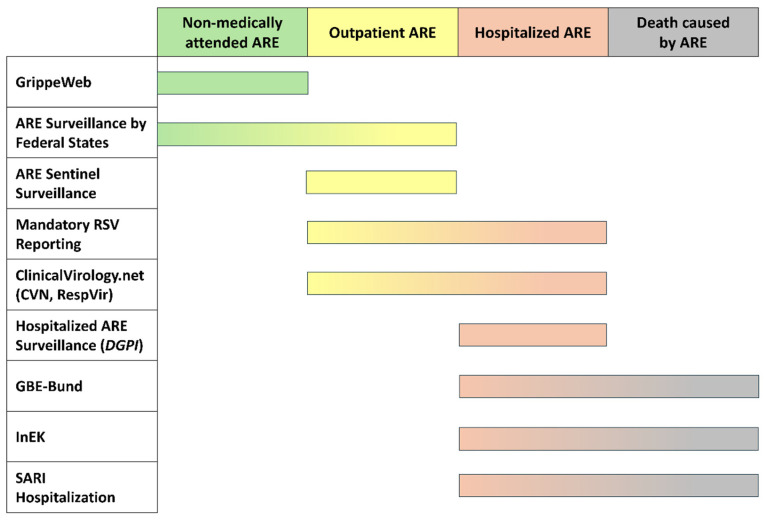
Surveillance systems and corresponding levels of disease severity captured (Non-medically attended ARE; Outpatient ARE; Hospitalized ARE; Death caused by ARE). Abbreviations: RSV = respiratory syncytial virus, ARE = acute respiratory disease, SARI = severe acute respiratory infections, DGPI = German Society for Pediatric Infectiology.

Information on RSVI in Germany is collected, analyzed, and presented from various sources and in various formats, with each of the different surveillance systems coming with specific strengths and weaknesses. RSV episodes may be captured in distinct surveillance systems contingent upon clinical severity (Figure 4). Disease progression can result in the same episode being sequentially documented in multiple data repositories.

Surveillance efforts by the DGPI and Clinical Virology Net (RespVir) focus on recording the number and percentage of positive tests and on promptly disseminating results. This approach offers valuable, near real-time insights into current epidemiological dynamics across a spectrum of pathogens, supporting healthcare professionals in allocating short-term resources.

Sample-based surveys with a high degree of representativity, such as the RKI ARE Sentinel Surveillance and ICOSARI programs, can estimate national case numbers.

RSV incidence rates are of greatest interest to determine the clinical, but also the economic burden of disease. The GBE-Bund system and InEK, which compile RSVI hospitalization cases from administrative data, stand out for their automated recording during the processing of DRG-based reimbursement claims. Their strength lies in their coverage, resulting from the comprehensive inclusion of almost all German hospitals, thus minimizing potential bias associated with systems that rely on smaller population samples that may be less representative. However, Cai et al. showed a high specificity of RSV-specific ICD-10 codes (99.8%), but very poor sensitivity (6%) for Germany in a comparison of ICD-10 codes with virological data [23]. Thus, data on RSV incidence rates from public databases relying on RSV-specific ICD-10 codes must be assumed to be an underestimation of the actual burden of disease—for adults to a much greater extent than for children [48,49]. This coding bias is likely due to diagnostic testing for RSV not being regularly conducted, especially in adult and senior patients presenting at hospitals with respiratory symptoms. Reasons are likely a lack of disease awareness and the absence of an efficient and specific antiviral therapy [50]. Improvement in the RSV testing of adults has been observed since the introduction of influenza/RSV combination PCRs, which revealed a substantial number of previously unrecognized RSV infections in adults.

PCR testing is considered the most sensitive test in both pediatric and adult patients. Adults and seniors, however, often exhibit lower viral loads during RSV infection compared to children [51], resulting in reduced test positivity rates and underreporting in older age groups [52]. Notably, no consistent correlation has been observed between viral load and disease severity [53]. Of importance for diagnostic practice, sensitivity can be substantially improved by testing multiple specimen types—such as saliva or sputum—in addition to nasopharyngeal swabs [52,54,55]. A recent modeling study from Spain confirms that the use of RSV-specific ICD-10 codes alone leads to an underestimation of the real burden of disease. Rates estimated through modeling were 1.33 times higher than those based on RSV-specific codes for infants. For adults aged 18–79 years, the estimated rates were 6–8 times higher than those based on RSV-specific codes, and for the elderly ≥80 years, underestimation based on ICD-10 codes alone was as high as 16 times [56]. The authors of a prospective surveillance study conducted between 2021 and 2023 in Thuringia also concluded that RSV-specific ICD-10 codes were not suitable for estimating the burden of RSV-pneumonia, as only a third of RSV-pneumonia cases were documented as such with RSV-specific ICD-10 codes [57].

A recent study analyzed nationwide data on RSV-specific ICD-10-coded hospitalizations from Germany and showed RSV hospitalization incidence rates between 0.1 and 11.08 per 100,000 persons per year between 2010 and 2018 in adults ≥60 years [16]. Polkowska-Kramek et al. model incidence rates that are at a minimum 21 times higher. In their analysis of statutory health insurance data from Germany, incidence rates of RSV-associated respiratory, cardiovascular, and cardiorespiratory hospitalizations in adults were modeled for the years 2015 to 2019 [17]. They report RSV-attributable respiratory hospitalization rates between 236.4 and 362.8 per 100,000 person-years in adults ≥60 years of age. According to this modeling study, incidence rates of RSV-attributable hospitalizations may actually be as high as 911.5 per 100,000 person-years when taking into account that in addition to respiratory disease, RSV can cause exacerbation of cardiological conditions [8,9,11], even though it may not have been diagnosed as such [17]. The above-mentioned prospective surveillance study from Thuringia showed an RSV-related ARI hospitalization incidence among those ≥60 years of 401.6/100,000 [58]. In a recent systematic literature review and meta-analysis of 21 studies, acute RSV-related respiratory infections in adults of 60 years and older in high-income countries were estimated at a rate of 1620 per 100,000 and the hospitalization incidence for RSVI was estimated at 150 per 100,000. The in-hospital case fatality rate was high at 7% [59].

Several studies suggest that a significant degree of under-ascertainment may occur in many epidemiological studies on RSVI [60,61]. Li et al. [60] examined the RSV-associated acute respiratory infection hospitalization burden in older adults in high-income countries. Using a two-step framework that incorporated empirical data on the RSV detection proportion of different clinical specimens and testing approaches as well as their statistical uncertainty, they estimated that the pooled hospitalization rate more than doubled from 157 per 100,000 (95%CI 98–252) for adults aged ≥ 65 years to 347 per 100,000 (95%CI 203–595) after accounting for under-ascertainment. The in-hospital case fatality rate of RSV was estimated at 6.1% (3.3–11.0).

Obviously, to unequivocally diagnose RSVI, laboratory tests must be done in the first place. Rozenbaum [62] showed that in a sample of 937 US hospitals, in adults of 65 years or more hospitalized for LRTI, testing for RSVI was conducted only infrequently, suggesting that a significant number of RSVIs remain undiagnosed. However, one positive side effect of the COVID-19 pandemic is the notable increase in the use of multiplex assays screening for respiratory viruses such as SARS-CoV-2, Influenza A and B, and RSV simultaneously. A recent study comparing German hospitalized RSV cases among adults 60+ between pre- and post-pandemic seasons (2019/20 vs. 2022/23) suggests an underdetection of RSV of up to sevenfold, which was revealed through the increase in testing in adults [63].

## 6. Conclusions and Outlook

The limited capacities of hospitals in Germany demand a critical ascertainment of the RSV burden of disease to allow the efficient management of medical care. Multiple initiatives contribute in different ways to RSVI surveillance. The recent implementation of a nationwide reporting mandate for RSVI applying standardized and comparable methods of assessment and analysis is anticipated to further enhance the quality of RSVI surveillance. Due to limitations regarding testing strategies and high-sensitivity diagnostic tools, particularly in the outpatient setting, infection rates deduced by the present surveillance systems in Germany may not completely reflect the real infection dynamics and may introduce bias by underestimation. Especially in adults, both early awareness of RSV-associated symptoms of the upper and lower respiratory tract and consistent testing must be strengthened, as they are key to accurately assessing the individual risk and societal burden of RSVI. It remains to be evaluated whether greater standardization of diagnostics and consistent demographic stratification could enhance the accuracy of national surveillance systems. Accurate and up-to-date epidemiological data on RSVI are imperative for effectively allocating healthcare resources and estimating the clinical, societal, and economic burden of the disease.

For clinicians, our findings highlight the importance of understanding the scope and limitations of different RSV monitoring systems. Awareness of the variability in timeliness and accuracy among data sources can improve the interpretation of local infection dynamics and help contextualize clinical decision-making. Reliable access to epidemiological information is particularly valuable for anticipating seasonal surges, optimizing patient management strategies, and informing preventive measures such as prophylaxis or vaccination.

From a research perspective, the review underscores the need for the harmonization and integration of existing monitoring systems. Enhanced surveillance frameworks could enable more robust epidemiological modeling, facilitate the evaluation of preventive and therapeutic interventions, and ultimately contribute to a deeper understanding of RSV’s impact within the broader spectrum of respiratory infections.

## Figures and Tables

**Table 4:** Links to surveillance systems/databases. All listed links lead to publicly accessible data or data visualizations.

	Surveillance System/Database	Short Description	Access to Data	Access to Visualization
RKI	Mandatory RSV reporting	Laboratory-confirmed RSV cases reported under the Infectious Disease Act; aggregated data published by RKI, publicly accessible.	SurvStat https://survstat.rki.de/Default.aspx (last accessed on 14 October 2025).	Infektionsradar https://infektionsradar.gesund.bund.de/de (last accessed on 14 October 2025).
	GrippeWeb	Online self-reporting of acute respiratory symptoms; used to estimate incidence; aggregated data available on RKI website.	Weekly reports https://edoc.rki.de/handle/176904/39 GitHub https://github.com/robert-koch-institut/GrippeWeb_Daten_des_Wochenberichts (both accessed on 14 October 2025).	Dashboard GrippeWeb https://public.data.rki.de/t/public/views/ARE-Dashboard/BevoelkerungGrippeWeb-Inzidenzen (last accessed on 14 October 2025).
	ARE sentinel surveillance	Weekly reports from GPs and pediatricians on ARE and influenza-like illness; aggregated data published by RKI.	Weekly reports https://edoc.rki.de/handle/176904/39 GitHub https://github.com/robert-koch-institut/ARE-Konsultationsinzidenz (both accessed on 14 October 2025).	RKI Diagrams https://influenza.rki.de/Diagrams.aspx Dashboard surveillance https://public.data.rki.de/t/public/views/ARE-Dashboard/Arztpraxen (both accessed on 14 October 2025).
	SARI hospitalization	ICD-10-based hospital surveillance for severe respiratory infections; anonymized data from ~70 hospitals; aggregated results publicly available.	Weekly reports https://influenza.rki.de/Wochenberichte.aspx (last accessed on 14 October 2025). GitHub https://github.com/robert-koch-institut/ARE-Konsultationsinzidenz (last accessed on 14 October 2025).	Dashboard SARI in hospitals https://public.data.rki.de/t/public/views/ARE-Dashboard/Krankenhaeuser (last accessed on 14 October 2025).
	GBE-Bund	Federal health reporting system compiling official health data; reports and indicators publicly accessible.	”Respiratory-Syncytial-Viren” „Diagnosedaten der Krankenhäuser nach Behandlungsort” https://www.gbe-bund.de/gbe/ (last accessed on 14 October 2025).	-
	InEK	Collects hospital performance and cost data for DRG reimbursement; aggregated data are publicly accessible via InEK data browser.	InEK Datenbrowser https://datenbrowser.inek.org/ (last accessed on 14 October 2025).	-
	Hospitalized ARE surveillance (DGPI)	Registry of hospitalized children with ARE (RSV, influenza, COVID-19); data visualization publicly available.	-	Surveillance
				• 2021/22 https://dgpi.de/rsv-survey-update/ • 2022/23 https://dgpi.de/awi-erfassung-update/ • 2023/24 https://dgpi.de/awi-erfassung-update-2023-2024/ • 2024/25 https://dgpi.de/awi-erfassung-update-2024-2025/ (all accessed on 14 October 2025).
	ClinicalVirology.net (CVN, RespVir)	Aggregates data on lab-confirmed respiratory pathogens (positive/negative, notifiable/non-notifiable) from ~60 labs in Germany, Austria, and Switzerland; trends are publicly available.	Upon request.	Dashboard https://www.arcgis.com/apps/dashboards/2a29f2bebc524c67b6250b64beea12bf (last accessed on 14 October 2025).

Abbreviations: RSV = respiratory syncytial virus, RKI = Robert Koch Institute, ARE = acute respiratory disease, SARI = severe acute respiratory infections, DGPI = German Society for Pediatric Infectiology.

## Abbreviations

The following abbreviations are used in this manuscript:

AG	Arbeitsgruppe (working group)
ARE	Akute Respiratorische Erkrankung (acute respiratory illness)
ARI	Acute Respiratory Infection
BIS	Bayern Influenza Sentinel
CFR	Case Fatality Rate
CVN	Clinical Virology Network
DGPI	Deutsche Gesellschaft für Pädiatrische Infektiologie (German Society for Pediatric Infectious Diseases)
DRG	Diagnosis-Related Groups
GBE	Gesundheitsberichterstattung (health reporting system)
GfV	Gesellschaft für Virologie (German Society for Virology)
GP	General Practitioner
LRTI	Lower Respiratory Tract Illness
LUA	Landesuntersuchungsanstalt (State Institute for Investigation)
NPI	Non-Pharmaceutical Interventions
PCR	Polymerase Chain Reaction
RKI	Robert Koch Institut
SARI	Severe Acute Respiratory Infections
STIKO	Ständige Impfkommission (Standing Committee on Vaccination)
